# EEG Correlates of Central Origin of Cancer-Related Fatigue

**DOI:** 10.1155/2020/8812984

**Published:** 2020-12-11

**Authors:** Didier Allexandre, Dilara Seyidova-Khoshknabi, Mellar P. Davis, Vinoth K. Ranganathan, Vlodek Siemionow, Declan Walsh, Guang H. Yue

**Affiliations:** ^1^Center for Mobility and Rehabilitation Engineering Research, Kessler Foundation, West Orange, NJ, USA; ^2^Department of Physical Medicine & Rehabilitation, Rutgers New Jersey Medical School, Rutgers University, Newark, NJ, USA; ^3^The Harry R. Horvitz Center for Palliative Medicine, The Taussig Cancer Center, The Cleveland Clinic, Cleveland, OH, USA; ^4^Taussig Cancer Center, The Cleveland Clinic, Cleveland, OH, USA; ^5^Geisinger Medical Center, Danville, PA, USA; ^6^Department of Biomedical Engineering, Lerner Research Institute, Cleveland Clinic, Cleveland, OH, USA; ^7^Department of Supportive Oncology, Levine Cancer Institute, Carolinas HealthCare System, Charlotte, NC, USA; ^8^Center for Supportive Care and Survivorship, Carolinas HealthCare System, Charlotte, NC, USA; ^9^School of Medicine, University of North Carolina at Chapel Hill, Charlotte, NC, USA

## Abstract

The neurophysiological mechanism of cancer-related fatigue (CRF) remains poorly understood. EEG was examined during a sustained submaximal contraction (SC) task to further understand our prior research findings of greater central contribution to early fatigue during SC in CRF. Advanced cancer patients and matched healthy controls performed an elbow flexor SC until task failure while undergoing neuromuscular testing and EEG recording. EEG power changes over left and right sensorimotor cortices were analyzed and correlated with brief fatigue inventory (BFI) score and evoked muscle force, a measure of central fatigue. Brain electrical activity changes during the SC differed in CRF from healthy subjects mainly in the *theta* (4-8 Hz) and *beta* (12-30 Hz) bands in the contralateral (to the fatigued limb) hemisphere; changes were correlated with the evoked force. Also, the *gamma* band (30-50 Hz) power decrease during the SC did not return to baseline after 2 min of rest in CRF, an effect correlated with BFI score. In conclusion, altered brain electrical activity during a fatigue task in patients is associated with central fatigue during SC or fatigue symptoms, suggesting its potential contribution to CRF during motor performance. This information should guide the development and use of rehabilitative interventions that target the central nervous system to maximize function recovery.

## 1. Introduction

Cancer-related fatigue (CRF) is defined as “a distressing, persistent, subjective sense of physical, emotional, and/or cognitive tiredness or exhaustion related to cancer and/or cancer treatment that is not proportional to recent activity and interferes with usual functioning” [1]. CRF is the most frequently reported (30%-90%) and undertreated symptom while having the greatest adverse influence on quality of life (both during and following treatment) of all cancer symptoms [1–4].

Fatigue in cancer patients is multifactorial and may be influenced by several demographic, medical, psychosocial, behavioral, and biological factors [3, 5]. However, fatigue can still persist after ruling out comorbid, environmental, or social contributing factors, pointing to an intrinsic biological mechanism of CRF [5]. The biological etiology of CRF is not fully understood and is still the subject of active research. Cancer and its treatment have been associated with abnormal immune and inflammatory responses and hypothalamic-pituitary-adrenal axis dysregulation causing neuroendocrine alteration and metabolic and mitochondrial cellular impairment [2, 4, 5]. Ultimately, they are hypothesized to affect the central and peripheral nervous systems with alterations in neural processes and regulations, generating fatigue and other behavioral changes [4, 5].

No unifying hypothesis has been convincingly developed to explain CRF etiology. Difficulty in defining and assessing CRF, symptom complexity, variability in expression and severity, and population heterogeneity presents unique challenges in advancing our knowledge of CRF. Physiological studies of motor fatigue may provide more objective information and additional insights into understanding the mechanisms of CRF. This is particularly relevant given the evidence supporting exercise as an effective approach for lessening CRF [6]. Our research in recent years has shown that CRF patients fatigue earlier than healthy controls during a prolonged submaximal muscle contraction [7, 8]. The relative contribution of peripheral fatigue at the muscle vs. central fatigue at the level of the brain or spine can be evaluated by measuring the twitch force (TF), an inverse measure of muscle reserve. TF is the amount of force generated by supramaximal stimulation of muscle or associated nerve at rest or during a sustained contraction; the lower the TF, the more fatigued the muscle. Interestingly, the TF and other myoelectric measures of muscle function indicated that the muscle at the end of the task was less fatigued in CRF than healthy controls, suggesting a greater amount of central fatigue [7–10]. Central fatigue in motor performance, defined as loss of voluntary activation of muscle, is complex and multifactorial [11–13]. It may arise at spinal and/or supraspinal levels from an increased inhibitory input, decreased motoneuron firing and excitability, or suboptimal cortical drive [11, 14]. One or more of these factors could be examined to better understand CRF.

Neuroimaging studies in healthy populations can provide insight into the supraspinal modulation of motor fatigue [15–19]. Neuroimaging findings have also helped monitor brain activities under fatigue conditions in clinical populations such as chronic fatigue syndrome [20, 21] and multiple sclerosis [22–25]. Similarly, functional [26–28] and structural [29] brain changes have been correlated with fatigue symptoms in cancer suggesting a potential cortical origin. However, no CRF study has examined brain signals correlating with fatigue during a physical fatigue task. To further support and understand the neural mechanisms of the central origin of CRF, we analyzed and compared EEG data collected between cancer patients and healthy controls during a motor fatigue task activity in a prior research project [7]. A greater understanding of the neural plastic changes occurring during the disease process will help guide the development and use of effective rehabilitative interventions to improve CRF.

## 2. Materials and Methods

### 2.1. Experimental Protocol

After participants completed the BFI, the maximum elbow flexion force (MEF) and handgrip strength were measured from the right dominant arm/hand. Participants were then asked to do the sustained contraction (SC) fatigue task by maintaining an isometric elbow flexion at 30% MEF until exhaustion (defined as a failure to maintain 30% MEF for >5 seconds). The M-wave, i.e., maximum electric stimulation-evoked compound muscle action potential, from the brachioradialis, one of the elbow flexors, was recorded before and after the SC by superficially stimulating the radial nerve on the lateral side of the upper arm. The evoked twitch force (TF) was acquired at rest (TF_pre_) before the SC, at 30-second intervals during the SC, and at rest after the SC (TF_post_) by stimulating the biceps brachii muscle with maximal intensity. The post to pre twitch ratio (TF_ratio_ = TF_post_/TF_pre_) was then computed; lower value < 1.0 indicates more muscle fatigue, i.e., peripheral fatigue at time of exhaustion. High-density EEG was also continuously recorded before, during, and after the SC. The MEF force was measured again immediately after the SC. Detailed procedures and results for all measures except brain activity (EEG) can be found in [7].

### 2.2. EEG Measurements

**Figure 1:**
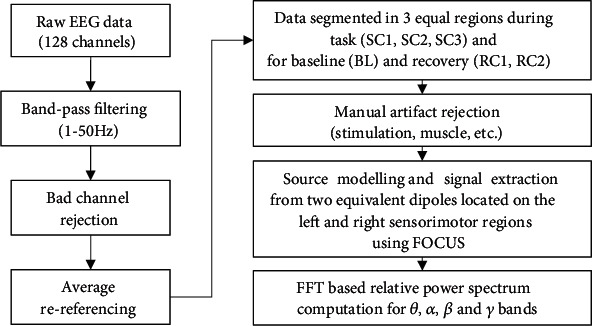
EEG processing pipeline.

Brain signals were recorded using a high-density 128 channel EEG data acquisition system from Electrical Geodesics, Inc. (Eugene, OR, USA) with impedance kept below 10 Kohms for motor regions. EEG signals were amplified (×75,000), band-pass filtered (0.1-100 Hz), and digitized (500 sample/s) using Neuroscan (Compumedic NeuroScan, Charlotte, NC). As illustrated in Figure 1, the EEG data were processed as follows. The data were band-pass filtered (1–50 Hz), and average referenced after noisy or high impedance channels were removed. Data were visually inspected to remove eye blinks, muscle, and movement artifacts. In particular, the experimental protocol involved electric stimulation-evoked twitch force of the biceps brachii, a major elbow flexor muscle about every 30 s during the SC to investigate the central vs. peripheral progression of fatigue (see [7] for details). These stimulations created large transient electrical and muscle artifacts typically lasting a few seconds. Typically, 10 to 30% of the data were removed throughout the fatigue task. Postprocessing and data analysis were then performed (1) at baseline rest, i.e., before the SC (BL), (2) during the SC, and (3) during the recovery period just after the SC (RC1) and 2 min later (RC2). The duration of the SC was further divided into 3 equal segments (based on signal duration before artifacts rejection) corresponding to the beginning, middle, and end of the SC (SC1, SC2, and SC3) to study fatigue progression [7].

This processed data was then used to extract the source activity waveforms of two equivalent brain dipoles representing activities in the left (LH) and right hemisphere (RH) brain regions associated with contralateral and ipsilateral sensorimotor cortices, by applying the FOCUS method and toolbox [30, 31] from the Brain Electrical Source Analysis (BESA, version 5.1 MEGIS) software [32]. The FOCUS method essentially removes the volume conduction effect on the recorded data by modelling brain source contributions to scalp activities using spatial deconvolution. Source localization was modelled as two independently time varying and spatially stationary equivalent dipoles with directions perpendicular to the scalp surface under electrodes C3 and C4, using a simplified spherical head model. Frequency power (nAm^2^) of the LH and RH source activity was then computed for *theta* (4-8 Hz), *alpha* (8-12 Hz), *beta* (12-30 Hz), and *gamma* (30-50 Hz) bands using Fast Fourier Transform (FFT). Given the high within and between subject variability in total power, results in each band are presented as relative power to the total power in each time segment for each participant.

### 2.3. Statistical Analysis

Between-group comparisons of BFI and participants' demographics were performed using simple *t*-test for continuous and Pearson's Chi-square for categorical data. Linear mixed effect regression models were used to model EEG data for each frequency band and each hemisphere. To confirm model validity, residuals were plotted to verify distribution normality. We used *F*-tests (type 3 tests) to evaluate the effect of group, time, and their interaction (denoted by “Group,” “Time,” and “Time^∗^Group,” respectively). Whenever significance was observed, post hoc contrast analyses were done for (1) within group (time effect with respect to baseline), (2) between-group comparisons for each time point, and (3) group by time interaction. The latter was done from baseline (BL) to end of contraction or recovery (SC3, RC1, and RC2) or from beginning to end of the SC (SC3 vs. SC1) to study the effect of and recovery from fatigue during contraction. Finally, whenever this post hoc analysis revealed a significant group by time interaction, we investigated the relation between EEG frequency power changes and measure of perceived fatigue (BFI scores), endurance (SC duration), and central vs. peripheral fatigue (twitch force ratio) by performing a Pearson's correlation analysis. To ensure that the correlation was not artificially driven by large group differences, we checked that the correlation remained with CRF only. To avoid spurious results (due to lack of normality or presence of outlier), results were checked against nonparametric Spearman's correlation and recomputed without outliers if necessary. Given the exploratory nature of the study, no adjustment for multiple comparisons was made.

## 3. Results

### 3.1. Participants

Sixteen patients with stage 3 or 4 solid cancers (lung, breast, and/or gastrointestinal cancer) referred to palliative medicine, and 16 age- and gender-matched healthy volunteers were recruited to the study. Eligible cancer patients had not had chemotherapy, radiation therapy, or surgery in the preceding 4 weeks. Detailed clinical and demographic information about the study participants is available elsewhere [7].

Most cancer patients had significant cancer-related fatigue (CRF) determined by the brief fatigue inventory (BFI) [33]. Thirteen of the 16 patients had moderate to severe levels of fatigue with scores ≥ 4. In comparison, all healthy controls had mild levels of fatigue with score < 4. Inclusion criteria was as follows: fatigue not caused by another factor such as anemia or depression, hemoglobin levels ≥ 10 g/dL, not on psychostimulants or antidepressants, not depressed based on the medical record and self-report assessment to the validated screening question “are you depressed?” [34], cancer − related weight loss by history < 10%, no cognitive impairment, severe polyneuropathy, amyotrophy, myasthenic syndrome determined clinically, or significant pulmonary compromise as defined by oxygen dependence. The study was approved by Cleveland Clinic's Institutional Review Board, where the study was performed.

### 3.2. Population Demographics and Fatigue Profile

There was no statistical difference between CRF and the control groups in age (mean ± standard deviation of 61.7 ± 10.3 yrs for CRF vs. 55.3 ± 10.9 yrs for controls, *p* = 0.10), gender proportion (9/16 vs. 11/16 female patients, Pearson's Chi-square *χ*^2^(1.32) = 0.533, *p* = 0.46), and BMI (25.7 ± 5.3 kg/m^2^ vs. 28.8 ± 6.5 kg/m^2^, *p* = 0.16). Patients reported feeling more fatigued than controls at the time of measurement (BFI score of 5.0 ± 1.8 vs. 0.9 ± 1.0, *p* ≤ 0.001).

### 3.3. EEG Outcome

As previously reported, CRF participants felt fatigue and ended SC task earlier than controls [7]. Therefore, the total duration of the fatigue task and consequently each resulting EEG data segment SC1, SC2, and SC3 (before artifact rejection) were shorter in CRF and varied between participants (fatigue task duration was 313 ± 145 s for CRF vs. 529 ± 131 s for controls, *p* < 0.001).

Table 1 shows that the relative power analyzed using the linear mixed model changed significantly over time for both left (LH) and right (RH) hemispheres for the *theta* (4-8 Hz) and *gamma* (30-50 Hz) bands and the LH *beta* (12-30 Hz) band, but not for the *alpha* (8-12 Hz) band. Furthermore, group difference was significant for the LH and trended toward significance (*p* = 0.07) for RH *theta* band only. The group by time interaction showed significant effects for the LH (contralateral to the performing arm) for all 4 frequency bands and the *theta* band only for RH, with some trend toward significance (*p* = 0.08) for the RH *gamma* band.

**Figure 2:**
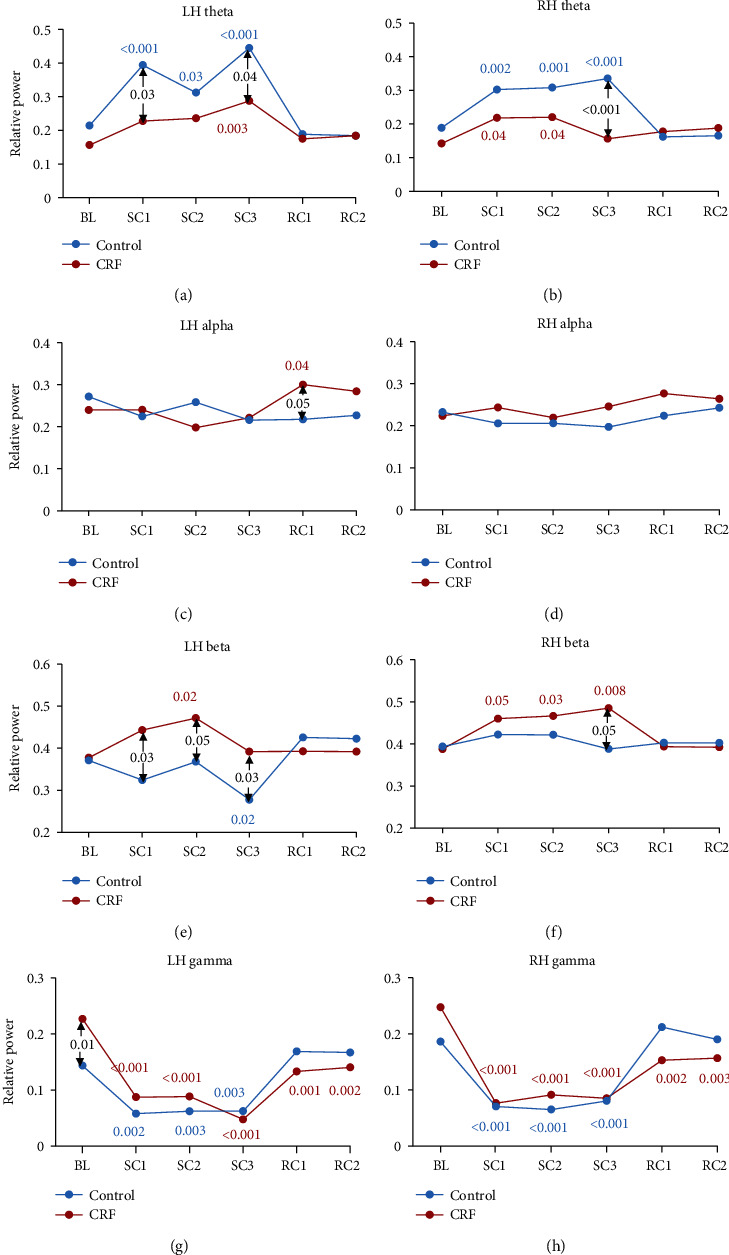
Relative EEG power change at each frequency band during the fatigue task for CRF (red) and healthy controls (blue) for the left (LH) and right (RH) hemispheres. Estimated marginal means are derived from the mixed linear model. Whenever significant, *p* values are shown in red for CRF and blue for controls for the within-group contrast analysis (compared to baseline) and in black for between-group contrast analysis. CRF: cancer-related fatigue; BL: pretask baseline; SC1, 2, and 3: beginning, middle, and end of the sustained contraction fatigue task, respectively; RC1 and 2: recovery period right after the end of the task and 2 min later.

The results of post hoc analyses using contrasts are demonstrated in Figure 2. In the within-group analysis, a *theta* band power (Figures 2(a) and 2(b)) increase was seen during the entire contraction (SC) for both hemispheres in both groups, but to a greater extent in controls, before returning to baseline during recovery. The between-group difference shows that the *theta* EEG band power was significantly greater in controls than CRF at the beginning of the SC (SC1) for the LH and end of the SC (SC3) for both hemispheres.

In contrast, no significant effect was observed for the *alpha* band (Figures 2(c) and 2(d)) except during recovery for the LH (RC1) where CRF showed a significant marginal increase in power (*p* = 0.04) and a significant group difference (*p* = 0.05).

The relative *beta* band (Figures 2(e) and 2(f)) power significantly increased for CRF in the RH during the SC for all three contraction periods (but marginally so for the LH, i.e., at SC2 only) before returning to baseline afterwards. In contrast, controls saw a marginal power decrease in the LH which only became significant at the end of the SC (SC3, *p* = 0.02) before returning to baseline (while no significant changes occurred in the RH). Between-group comparisons revealed that the EEG power at *beta* band differed significantly at all three SC periods for the left hemisphere and only at SC3 for the right hemisphere.

The within-group analysis also revealed that the *gamma* band (Figures 2(g) and 2(h)) power substantially decreased for both groups during the entire SC (*p* < 0.01) for both LH and RH, but did not return to prefatigue values in the CRF group after a 2min recovery period (RC2, *p* < 0.003). The between-group comparison shows that the relative *gamma* power was significantly greater at baseline in the CRF group than controls (*p* = 0.01).

The group by time post hoc analysis during the SC task (SC3 vs. SC1) reveals a trend toward significance in RH *theta* power (*t*(150) = −1.821; *p* = 0.07), where it increased in controls, but decreased in CRF.

### 3.4. Correlation Analyses

**Figure 3:**
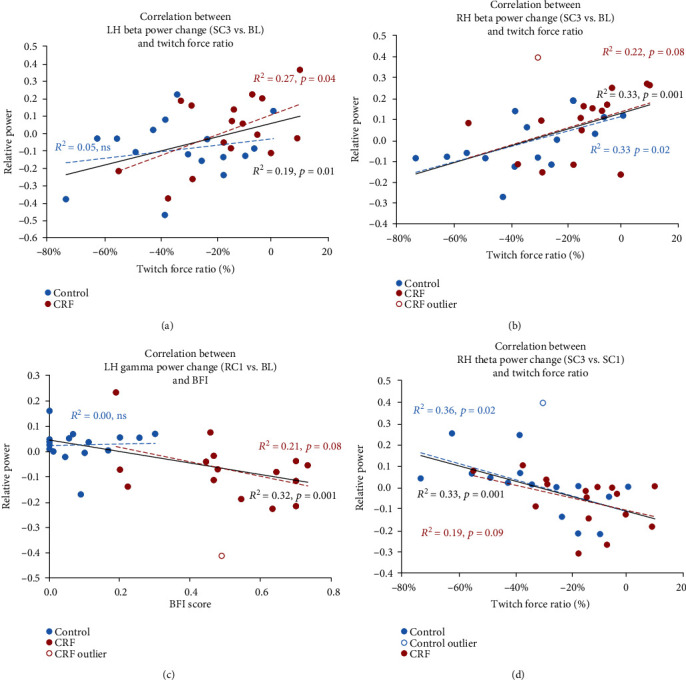
Linear correlation between (1) relative power change during the sustained contraction task (SC3 vs. SC1), at end of the task (SC3), or posttask (RC1) compared to baseline (BL) for CRF (red) or controls (blue); (2) twitch force ratio or BFI score. Linear fits are shown in black for the whole sample, blue for controls, and red for CRF. Outliers are shown in open circles; linear fitting was done without the outliers. CRF: cancer-related fatigue; BFI: brief fatigue inventory.

The group difference in LH *gamma* power at baseline (Figure 3) was not associated with any significant correlation with BFI.

Group by time effect from baseline to the end of SC (SC3) shows a significant interaction for RH *theta* (*p* = 0.01) and *beta* power (*p* = 0.005) and a trend toward significance for LH *beta* power. Subsequent correlation analysis revealed a significant positive relation between this LH and RH *beta* power change and twitch force ratio (Figures 3(a) and 3(b); *R*^2^ = 0.19, *p* = 0.01 and *R*^2^ = 0.33, *p* = 0.001, respectively, for the whole sample; Spearman *ρ*^2^ = 0.25 and *p* = 0.004 for the RH *beta* power when including the CRF outlier; *R*^2^ = 0.27, *p* = 0.04 and *R*^2^ = 0.22, *p* = 0.08 for CRF only). No correlation was found with BFI or task duration.

A significant group by time effect for postcontraction compared to baseline was also found for LH *alpha* (*p* = 0.006 for RC1 and *p* = 0.03 for RC2) and LH and RH *gamma* (*p* = 0.002 and 0.006 for RC1 and 0.005 and 0.029 for RC2, respectively). Subsequent correlation analysis showed a significant negative correlation between BFI and LH, but not RH *gamma* power change (Figure 3(c); *R*^2^ = 0.32 and *p* = 0.001 for the whole sample except for one CRF outlier and *R*^2^ = 0.21 and *p* = 0.08 for CRF only; Spearman *ρ*^2^ = 0.33 and *p* = 0.001 for the whole sample).

Finally, a significant group by time interaction was found for RH *theta* power change during the SC (SC1 to SC3) (*t*(150) = −1.821; *p* = 0.07), where it increased in controls, but decreased in CRF. This change significantly correlated with twitch force ratio (Figure 3(d); *R*^2^ = 0.33 and *p* = 0.001 for the whole sample excluding one control outlier and *R*^2^ = 0.36 and *p* = 0.02 for CRF; Spearman *ρ*^2^ = 0.48 and *p* < 0.001 for the whole sample), but not with BFI and task duration.

## 4. Discussion

Our prior research found that the inability of CRF to sustain a submaximal muscle contraction as long as healthy controls is largely the result of greater central fatigue at the spinal and/or cortical levels rather than at peripheral or muscle level [7–10]. Here, we analyzed EEG data collected during the motor activity to assess whether it would support the central origin of CRF and improve our understanding of the supraspinal origin of the symptoms.

The main findings of the current analysis are as follows: (i) the relative LH EEG *gamma* frequency (30-50 Hz) power at baseline is significantly greater for CRF than controls; (ii) the relative LH (and somewhat RH) *theta* (4-8 Hz) power is lower, and the *beta* (12-30 Hz) power is greater in CRF than controls during the sustained contraction (SC), (iii) and this elevated *beta* power correlates with the twitch force ratio, a measure of central fatigue at the end of the SC; (iv) in contrast to healthy controls, the relative LH and RH *gamma* and LH *alpha* (8-12 Hz) power did not fully recover back to baseline in CRF after the SC; (v) this lack of recovery for LH *gamma* power in CRF was correlated with the level of overall perceived fatigue (BFI).

A greater relative *gamma* band power at baseline may point to a potential marker of fatigue at rest in CRF. This is difficult to explain, however, as it did not correlate to BFI scores. Fatigue has been more commonly associated with *theta*, *alpha*, or *beta* bands in other pathologies, like chronic fatigue syndrome [20, 21, 35], multiple sclerosis [24, 36], and burnout patients for whom their *alpha* and/or *beta* frequency power correlated with subjective fatigue [37]. Perhaps the *gamma* band power only reflects a subjective feeling of fatigue, but does not relate to its severity or may relate to other cancer-related symptomologies.

During the SC, we observed a decrease in relative *gamma* band power for both CRF and controls. This is noteworthy as usually tonic muscle contractions are promoted by *beta* oscillations whereas changes in EEG *gamma* power occurs mainly during dynamic motor activities [38, 39], although alterations of the *gamma* band power have previously been observed during a short-duration SC [40]. It is unclear why *gamma* band power *decreased* as *gamma* wave *synchronization* (i.e., increase in its power) usually occurs during dynamic motor control task where it is thought to support visuomotor integration [38, 40, 41]. This would need further investigation.

More importantly, CRF shows abnormal lower *gamma* power in the LH and RH post-SC, as it failed to fully recover back to baseline (Figures 2(g) and 2(h)), suggesting that the altered brain signal by the fatiguing SC lingered longer in CRF than healthy controls. Furthermore, the amplitude of lack of recovery in the LH *gamma* power was negatively correlated with BFI in CRF. In other words, the more subjective fatigue, the less neural recovery from the motor fatigue indicated by the *gamma* band signal. Interestingly, the larger the abnormally elevated *gamma* band power at rest, the greater the lack of recovery (*R*^2^ = 0.6 and *p* < 0.001) for CRF, suggesting a close relation between persistent symptoms of fatigue, elevated EEG *gamma* power, and subsequent lack of recovery of the signal from motor fatigue induced by the SC. Further research is needed to fully understand the underlying physiological mechanisms at play.

Controls had a greater increase in relative *theta* band activity than CRF during the SC. This was present in the LH (Figure 2(a), SC1 and SC3) for the duration of the SC, but also in the RH (Figure 2(b), SC3) at the end of the SC, i.e., at the highest level of physical fatigue. Interestingly, the increase in RH *theta* power at the end compared to the beginning of the SC seems to be inversely correlated with the twitch force ratio (Figure 3(d)). This would suggest that the RH (and to some extent LH) *theta* power may be directly correlated to the level of muscle fatigue and therefore the relative level of peripheral vs. central fatigue, which was less in CRF [7–10]. It has been previously proposed that low-frequency neural signal synchronization spanning across *delta* and *theta* bands (<8 Hz) may entrain and coordinate distant brain regions to enhance information processing when task demands increase, as well as integrate motor commands and incoming visual-somatosensory information to improve motor performance [41–43]. Greater *theta* activity may thus be consistent with neuroimaging findings of greater cortical demands on a wider motor network during fatigue to maintain task performance and force output [15, 16, 44]. This increased cortical activity may represent either (1) an increase in nociceptive groups III and IV afferent input to sensorimotor cortex from muscles as they fatigue; (2) a greater cortical drive primarily from higher-order motor cortices, such as the prefrontal and premotor areas, to compensate for the loss of drive from the primary motor cortex which must increase motor unit recruitment and firing to maintain force production as the muscle fatigues; or (3) both [11, 14, 17–19]. In this framework, the lower *theta* activity increase in CRF may be a direct reflection of reduced afferent input and thus somatosensory processing due to lesser peripheral fatigue [7, 8] or may point to a central origin of fatigue as the inability to engage and coordinate distant brain regions to respond to the increase in task demand.

This proposed explanation of response failure to increased cortical demands or decreased afferent inputs associated with lower peripheral fatigue in CRF is also consistent with the observed low *beta* power in the LH (and some RH) and the absence of LH *beta* desynchronization seen in healthy controls. *Beta* desynchronization (power decrease) is believed to reflect the release of background cortical inhibition involved in gating motor commands and somatosensory inputs during movement execution [42, 45]. Greater *beta* desynchronization has been observed in submaximal sustained fatigue compared to a nonfatigue contraction task (40% vs. 5% of MVC) and is thought to reflect increased corticospinal output to maintain consistent force and increased afferent feedback [46]. This aligns with our observed linear relationship between *beta* power desynchronization at the end of the SC and twitch force ratio. The greater the peripheral fatigue (the lower the twitch force ratio), the greater the decrease in *beta* power. Depressed *beta* power during the SC in CRF may thus reflect (1) the inability to release background cortical inhibition (or increase its excitability) to promote motor output or (2) a reduction in afferent input and thus somatosensory processing from lower peripheral fatigue/higher central fatigue associated with earlier task failure.

Electrophysiology and neuroimaging fatigue studies have been performed in other populations. However, few have been conducted during a physical task making it difficult to compare our results to those in the literature. Elevated cortical resting state activity or reduced connectivity or efficiency in the low frequency bands (*theta* in particular) has consistently been reported in chronic fatigue syndrome (CFS) [35, 47–49], especially in the sensorimotor and frontal cortical regions. Similarly, more relative *theta* activity was reported during a fatiguing contraction task in CFS [21]. Like CFS, consistent with structural and functional connectivity loss from demyelination or other neural damage, fatigue in multiple sclerosis (MS) correlates with functional connectivity impairment especially in the frontal (such as SMA) and parietal (somatosensory cortex) cortical network, suggesting a critical role of sensory processing in modulating fatigue perception [24, 50]. This impaired connectivity might explain an increase in *beta* and *theta* bands and decrease in the *alpha* band resting state EEG activity—and reduced *alpha* band coherence—in the same regions compared to control [51, 52], even though their correlation with fatigue was not explored. This connectivity loss may explain a compensatory increase in cortical activity during simple motor tasks, not only in motor areas, but also in those involved in sensorimotor integration and attention, like prefrontal and parietal areas [36, 53–56]. In particular, Leocani et al. observed greater frontal *beta* desynchronization during movement preparation and reduced postmovement frontal and contralateral central *beta* synchronization (reflecting impaired disinhibition) in fatigued compared to nonfatigued MS patients and healthy subjects [36]; effects correlated with fatigue severity. This is consistent with the need for a greater compensatory central drive during a submaximal fatigue task [57]. This can result in a blunted response when task demands exceed compensation capacity like during a maximal fatigue task or more advanced stage of the disease [58, 59]. This overall greater activity seen both in MS and CFS during contraction (or rest) is the opposite of what we observed in CRF. This may suggest a different cortical response in CRF to a common underlying functional connectivity disruption or inefficiency. CRF may have limited cortical voluntary drive at the onset leading to greater central fatigue and early SC failure. This is consistent with some of our prior findings on the same experimental data [10, 60]. First the corticomuscular coupling, a measure of corticomuscular drive, was found to be already weaker in the first half of the task, i.e., before experiencing strong fatigue [60]. Second, there was no intrinsic pre- to postfatigue change in muscle property, including muscle electrical signals and force generation capability [10], suggesting that fatigue resulted from an inability of the central nervous system to fully activate the elbow flexor muscles in individuals with CRF. In contrast, CFS or MS patients need to exert greater cortical effort—as a compensatory mechanism—to maintain motor output (compared to healthy participants), consistent with greater perception of effort during a fatigue task in CFS [61] and MS [57, 59].

The analysis of our EEG data was limited to the sensorimotor regions around the central sulcus. Future efforts should look at neural correlates of CRF by studying brain activity and connectivity within the wide motor network regions, in particular the sensory and frontal cortical regions given their potential roles in supraspinal fatigue. To this end, advanced processing techniques, like independent component analysis or source imaging, have been successfully used to study fatigue in endurance cycling [62] and air traffic control [63]. In addition, corticomuscular coherence can study changes in brain to muscle drive or muscle to brain afferent inputs in CRF compared to healthy individuals [60]. Similarly, transcranial magnetic stimulation (TMS) on the spine and brain would also help investigate changes in brain excitability and the relative supraspinal and spinal contribution to central fatigue. These advanced and complementary processing techniques and modalities would also provide valuable information to help elucidate whether neural changes observed in CRF are a manifestation of less peripheral fatigue or a direct contributor to greater central fatigue.

Other limitations to the study include small sample size and lack of control for medications such as antibiotics, anticholinergics, corticosteroids, and opioids that may influence neuromuscular conduction. We did not screen for antibodies to acetylcholine receptors or voltage-gated calcium channels which would impair neuromuscular junction conduction.

## 5. Conclusion

Compared to healthy controls, CRF is accompanied by abnormal brain activities and modulations before and during a sustained fatiguing muscle contraction, which fail to fully recover right after the task. Some of those abnormal brain changes correlate with measure of subjective (BFI) and objective (twitch force ratio) fatigue. The specific neural changes in CRF would suggest that early fatigue and task failure may reflect an inability of the brain to respond to the increase in task demand or may be the result of decreased afferent inputs associated with lower peripheral fatigue in CRF. These results support our prior findings of central origin of CRF in motor performance, but also suggest supraspinal contribution to the symptoms, rather than at levels of the spinal cord motor nuclei or at the neuromuscular junction.

This information is critical to understand and help the development of effective rehabilitation. In particular, this would explain why relaxation as well as mild to moderate intensity exercise interventions including yoga, which have neuroprotective or promote neurogenesis and neuroplasticity, [64] have been found most effective at improving CRF [65–67]. Noninvasive brain stimulation such as transcranial direct current stimulation (tDCS) has shown to improve fatigue in neurological disease such as multiple sclerosis [68–70]. Its effectiveness should be similarly studied in CRF. Additional research is needed to confirm the supraspinal origin, as well as the specific brain regions or network connections most affected by CRF. This would guide the development of more effective rehabilitative interventions by helping to target specific brain regions or network to maximize functional recovery in CRF.

## Figures and Tables

**Table 1:** Linear mixed model type 3 *F*-tests to evaluate the effect of time, group, and their interaction for all four frequency bands and both left and right hemispheres.

		*Theta*		*Alpha*		*Beta*		*Gamma*	
		Left	Right	Left	Right	Left	Right	Left	Right
Time	*F*(5.150)	13.1	6.71	1.41	1.24	2.39	1.91	16.11	17.56
	*p*	**<0.001**	**<0.001**	0.22	0.29	**0.04**	0.1	**<0.001**	**<0.001**
Group	*F*(1.30)	4.58	3.62	0.19	0.96	1.52	0.54	0.21	0.001
	*p*	**0.04**	0.07	0.67	0.33	0.23	0.47	0.65	0.97
Group^∗^time	*F*(5.150)	2.61	4.15	3.36	0.63	3.31	1.39	2.74	1.98
	*p*	**0.03**	**0.001**	**0.007**	0.68	**0.007**	0.23	**0.02**	0.08

## Data Availability

The data used to support the findings of this study are available from the corresponding author upon request.
